# Supplementary material for: Stable maternal proteins underlie distinct transcriptome, translatome, and proteome reprogramming during mouse oocyte-to-embryo transition
Source: Genome Biol. 2023 Jul 13;24:166. doi: 10.1186/s13059-023-02997-8 (PMC10347836; doi:10.1186/s13059-023-02997-8)
Supplement: Supplementary file 1 — Additional file 1: Supplementary figure and figure legends. Figure S1. Validation of LC-MS/MS quantification ability in mouse oocytes. Figure S2. Evaluation of LC-MS/MS data quality in mouse oocytes and early embryos. Figure S3. Dynamic changes of RPF and mRNA in the mouse oocytes and early embryos. Figure S4. Correlation between protein and mRNA across developmental stages. Figure S5. Differentially expressed proteins and genes at each consecutive stage. Figure S6. Correlation between protein and RPF or mRNA for individual genes across developmental stages. Figure S7. Protein features related to the concordance between protein and RPF or mRNA. [file 13059_2023_2997_MOESM1_ESM.docx]

**
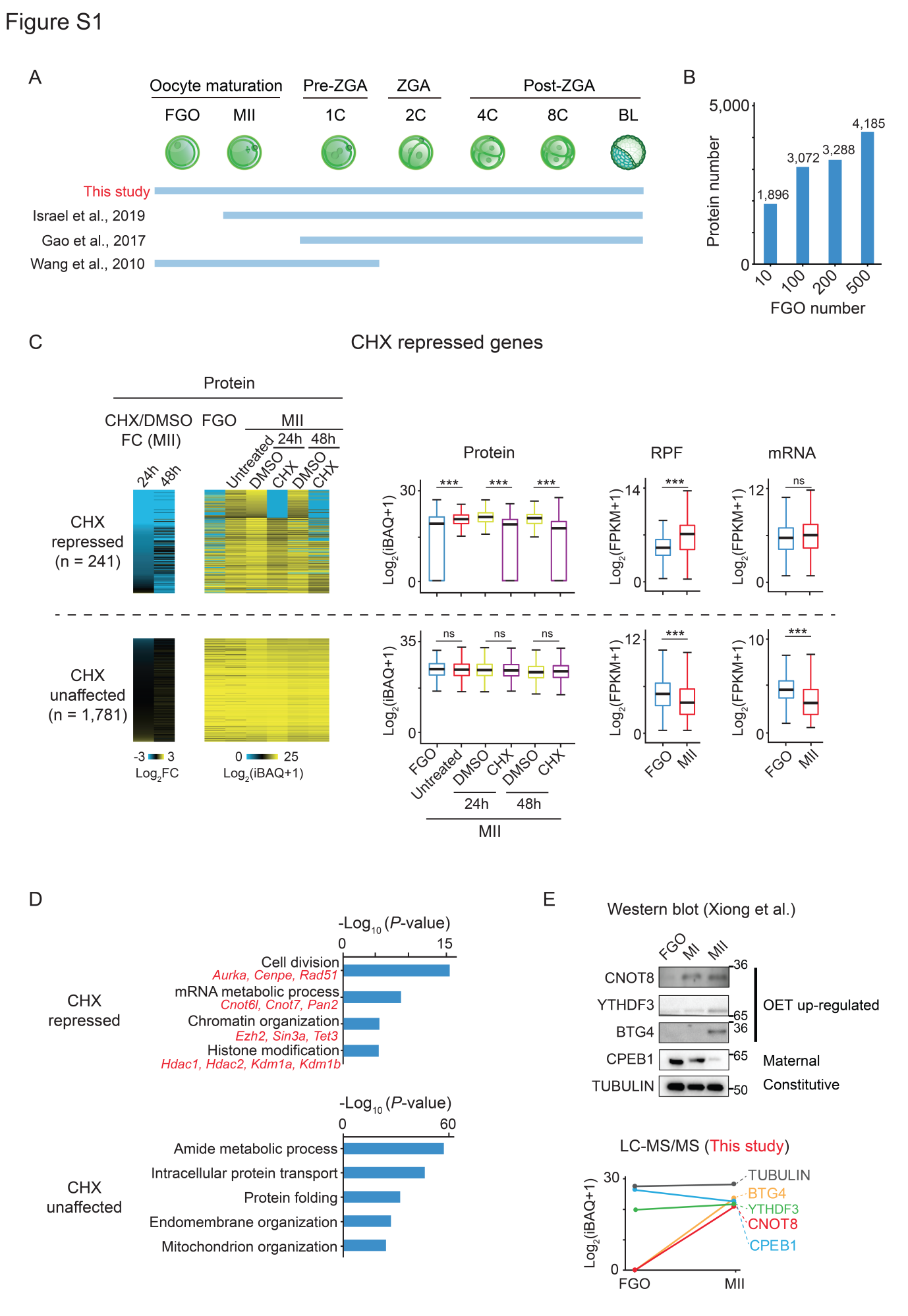
**

**Figure S1. Validation of LC-MS/MS quantification ability in mouse oocytes.**

**(A)** A schematic showing the developmental stages of oocytes and embryos covered in this study and the previous datasets.

**(B)** Detected protein numbers using varying numbers of input FGOs.

**(C)** Heat maps showing the protein fold changes (FC) and protein intensities in FGOs and MII oocytes treated with DMSO or CHX for 24 h or 48 h (left); box plots showing the protein, RPF, and mRNA levels in untreated FGOs and MII oocytes, and protein levels in MII oocytes treated with DMSO or CHX for 24 h or 48 h.

**(D)** Bar plots showing the enriched GO terms for CHX repressed and unaffected genes. The representative genes for each term are also shown.

**(E)** Protein levels in oocytes for five example genes quantified via Western blot in our previous study (top) or by LC-MS/MS from this study (bottom).

**
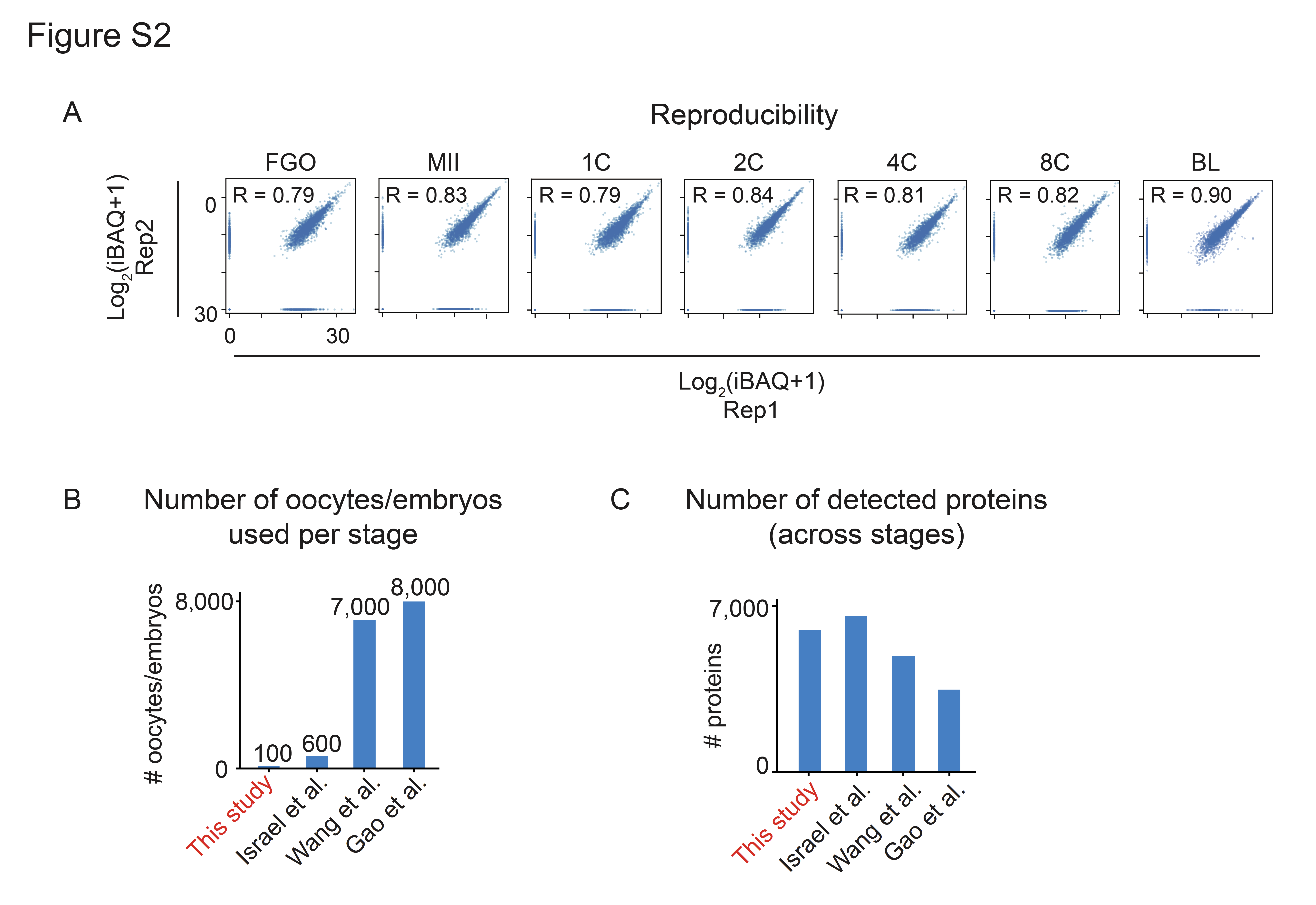
**

**Figure S2. Evaluation of LC-MS/MS data quality in mouse oocytes and early embryos.**

**(A)** Scatter plots comparing the protein intensity between biological replicates of LC-MS/MS data for each stage. The Spearman correlation coefficients are also shown.

**(B)** Bar plots showing the numbers of oocytes and embryos utilized in this study and the previous datasets.

**(C)** Bar plots showing the numbers of proteins detected in this study and the previous datasets.


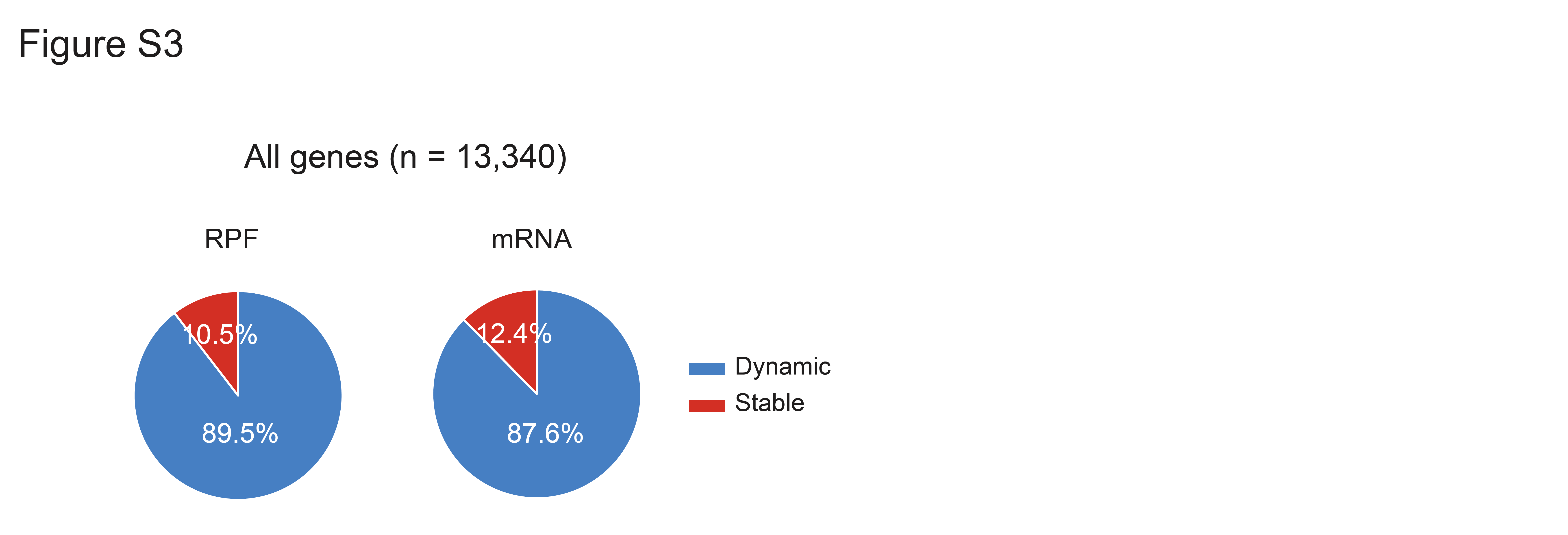


**Figure S3. Dynamic changes of RPF and mRNA in the mouse oocytes and early embryos.**

Pie charts showing the proportions of stably and dynamically regulated RPFs and mRNAs for all genes expressed in at least one stage (FPKM >= 1) (n = 13,340) of oocytes and early embryos with a coefficient of variation > 0.2 across stages.

**Figure S4. Correlation between protein and mRNA across developmental stages.**

**(A)** Heat map showing the Spearman correlation coefficients between pairwise protein and mRNA across developmental stages. The black boxes indicate the highly-correlated stages.

**(B)** Heat map of the Spearman correlation coefficients between protein changes and mRNA changes across developmental stages. The dashed lines represent synchronized (black), one-stage lagging (red), and two-stage lagging (blue) changes. Arrows indicate the highly-correlated transition stages.


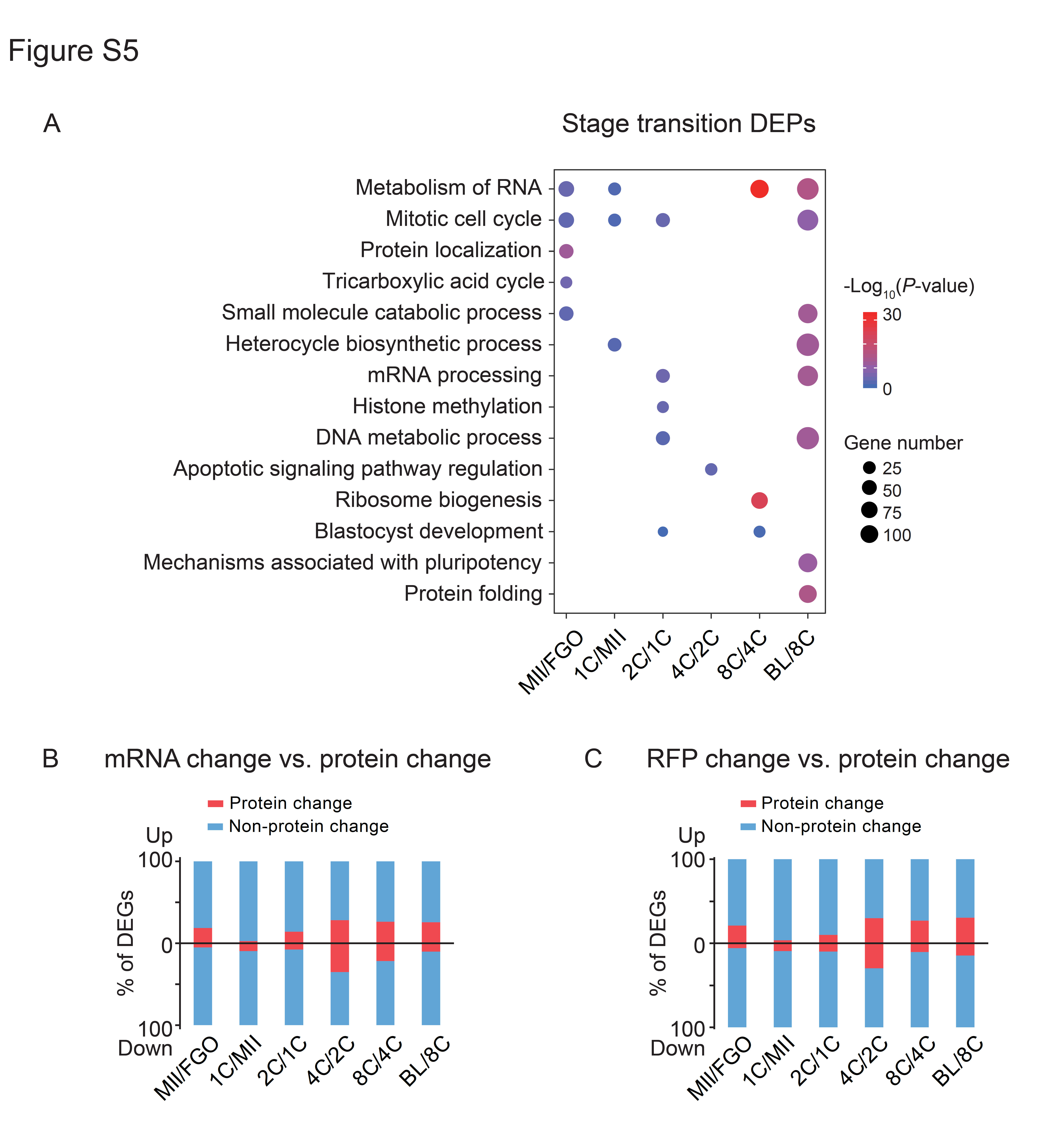


**Figure S5. Differentially expressed proteins and genes (mRNAs and RPFs) at each consecutive stage.**

**(A)** Bubble plot showing the enriched GO terms of DEPs at each transition between consecutive stages. The size of circle encodes gene number, and the color of circle indicates -log10(*P*-value).

**(B-C)** Bar plots showing the percentages of differentially expressed mRNAs **(B)** and differentially expressed RPFs **(C)** that coincided with substantial changes in proteins. As protein changes lag behind RNA changes, the DEPs of the current stage and the two following stages were all considered.


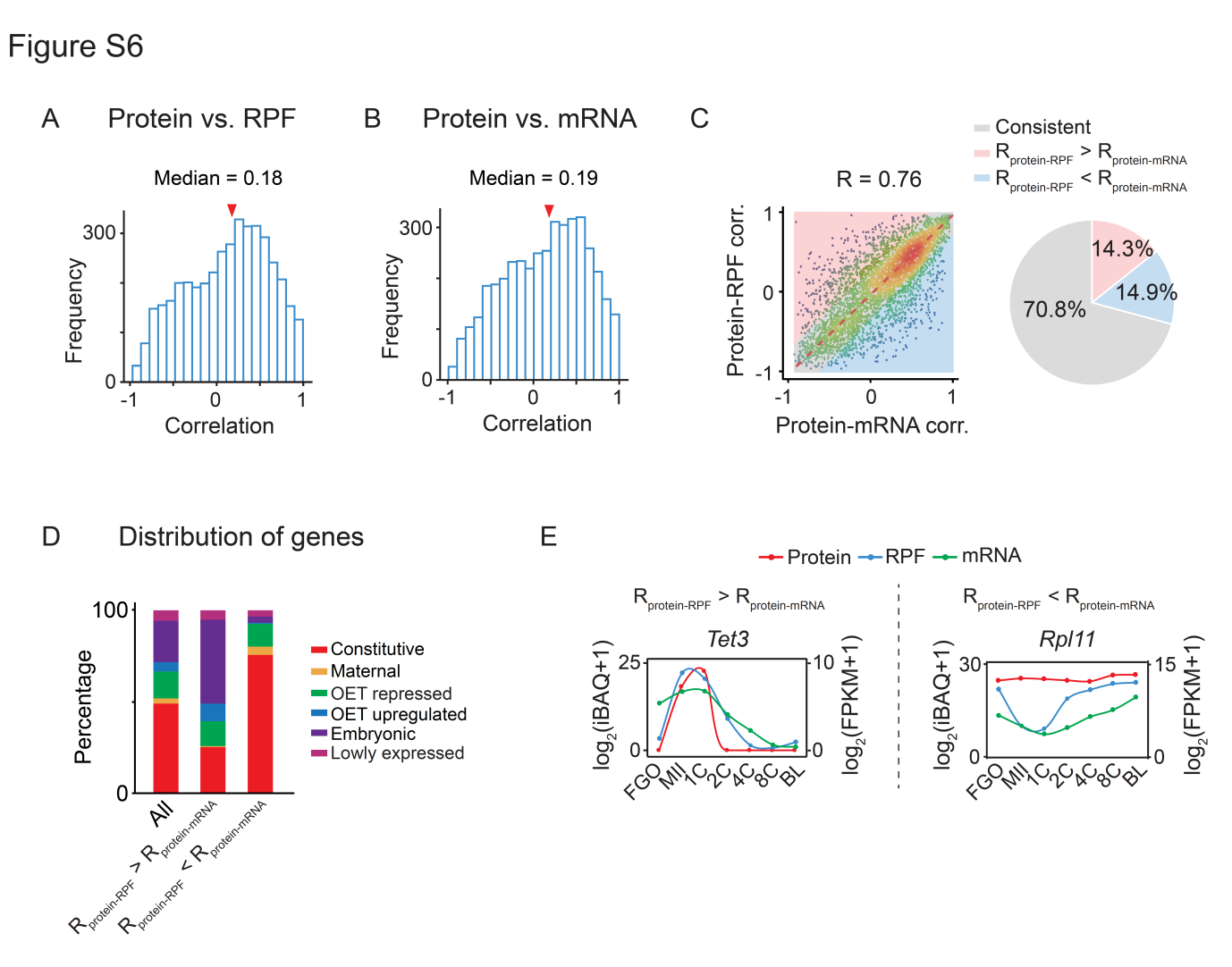


**Figure S6. Correlation between protein and RPF or mRNA for individual genes across developmental stages.**

**(A-B)** Histogram showing the Spearman correlation coefficients of protein-RPF (A) or protein-mRNA (B). The median correlations are also indicated.

**(C)** Left, scatter plot comparing protein-RPF and protein-mRNA Spearman correlation coefficients. Right, pie chart displaying the proportions of different groups. Grey shade, proteins correlated with RPFs and mRNAs to a similar degree (|$R_{protein-RPF}$ - $R_{protein-mRNA}$| < 0.3); pink shade, proteins correlated better with RPF than mRNA ($R_{protein-RPF}$ - $R_{protein-mRNA}$ > 0.3); blue shade, proteins correlated better with mRNA than RPF ($R_{protein-RPF}$ - $R_{protein-mRNA}$ < -0.3).

**(D)** Bar plot showing the distribution of different protein groups in “All” proteins and proteins correlated better either with RPF or mRNA.

**(E)** Line plots showing protein (red), RPF (blue), and mRNA (green) dynamics across developmental stages for representative genes from the $R_{protein-RPF}$ > $R_{protein-mRNA}$ group (left) or the$R_{protein-RPF}$ < $R_{protein-mRNA}$ group (right).

**
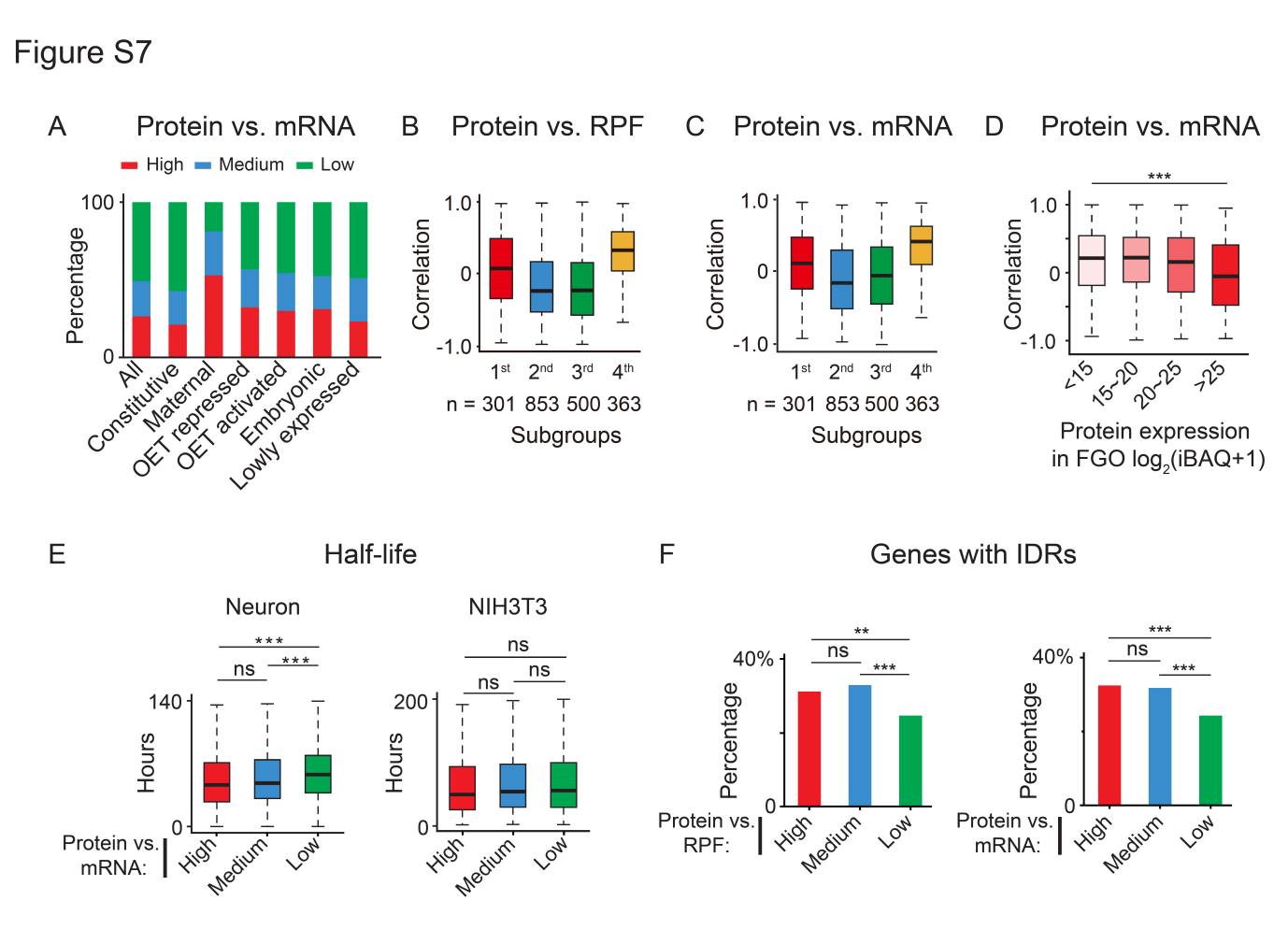
**

**Figure S7. Protein features related to the concordance between protein and RPF or mRNA.**

**(A)** Bar plots showing the percentage of proteins that showed high (R > 0.5), medium (R = 0.2-0.5), or low (R < 0.2) correlation (Spearman) with mRNA across the developmental stages for “All” proteins and different protein groups in Fig. 3A.

(**B-C**) Box plots showing the protein-RPF (**B**) and protein-mRNA (**C**) correlations for protein subgroups defined in Fig. 3A. n, the protein number in each subgroup.

**(D)** Box plots showing the protein-mRNA correlation based on the protein abundance in FGOs.

**(E)** Box plots showing protein half-lives determined in mouse embryo neuron or embryonic fibroblast cell line NIH3T3 in different protein-mRNA correlation groups.

**(F)** Bar plots showing the percentage of proteins containing intrinsically disordered regions (IDRs) for protein groups defined by protein-RPF (left) or protein-mRNA (right) correlations. The significance for all the above plots was calculated by the Wilcoxon rank-sum test (two-tailed). ***, *P*-values < 0.001; **, *P*-values < 0.01; ns, non-significant.
